# Morphology and Mitochondrial Lineage Investigations Corroborate the Systematic Status and Pliocene Colonization of *Suncus niger* (Mammalia: Eulipotyphla) in the Western Ghats Biodiversity Hotspot of India

**DOI:** 10.3390/genes14071493

**Published:** 2023-07-22

**Authors:** Shantanu Kundu, Manokaran Kamalakannan, Ah Ran Kim, Vishwanath D. Hegde, Dhriti Banerjee, Won-Kyo Jung, Young-Mog Kim, Hyun-Woo Kim

**Affiliations:** 1 Department of Marine Biology, Pukyong National University, Busan 48513, Republic of Korea; 2 Mammal and Osteology Section, Zoological Survey of India, M Block, New Alipore, Kolkata 700053, India; 3 Western Ghat Regional Centre, Zoological Survey of India, Kozhikode 673006, India; 4 Research Center for Marine Integrated Bionics Technology, Pukyong National University, Busan 48513, Republic of Korea; 5 Marine Integrated Biomedical Technology Center, National Key Research Institutes in Universities, Pukyong National University, Busan 48513, Republic of Korea

**Keywords:** small mammal, soricids, molecular systematics, phylogeny, diversification

## Abstract

**Simple Summary:**

The current study reassesses the systematic status of *Suncus niger* from its original geographical distribution in Southern India. Both morphology and mitochondrial genetic data clearly distinguish *S. niger* (endemic to India) from *Suncus montanus* (endemic to Sri Lanka) and substantiate the prior hypothesis of a distinct species. Furthermore, the present study adds new range extension, elevation records, and Pliocene colonization for this small mammal in the Western Ghats biodiversity hotspot. Given the matrilineal affinities between Indian and African lineages, and their recent transcontinental colonization, we believe that more in-depth genomic studies, including divergence time estimation, are crucial to clarify the global evolutionary trend of Soricomorphs.

**Abstract:**

The Indian highland shrew, *Suncus niger* (Horsfield, 1851), is the least studied soricid species from its original range distribution in Southern India, with several systematics conundrums. Following its discovery in 1851, the species was synonymized with *Suncus montanus* (Kelaart, 1850) (endemic to Sri Lanka) and subsequently identified as a separate Indian population. However, the systematic status of *S. niger* from topotype specimens in Southern India has yet to be determined through an integrated approach. Both taxonomy and mitochondrial genetic data (Cytochrome b and 16S ribosomal RNA) were used to re-examine the systematics of *S. niger*. The mtCytb gene clearly distinguished topotypic *S. niger* from other *Suncus* species, with high genetic divergences varying from 8.49% to 26.29%. Further, the Bayesian and maximum likelihood topologies clearly segregated *S. niger* from other congeners and corroborated the sister relationship with *S. stoliczkanus* with expected divergence in the late Pliocene (2.62 MYA). The TimeTree analysis also exhibits a strong matrilineal affinity of *S. dayi* (endemic to India) toward the African species. The current study hypothesizes that the ancestor of the soricids evolved in Africa and that genetic lineages were subsequently shifted by plate tectonic events that subsequently colonized different continents as distinct species during the late Miocene (Tortonian) to the Holocene era. In addition to the new range expansion and elevation records of *S. niger* in the Central Western Ghats, we propose that additional sampling across its distribution, as well as the use of multiple genetic markers, may be useful in determining the genetic diversity and population structure of this endemic species. The present study also recommends that more molecular data on the Soricomorphs lineages, and estimates of their divergence times, will shed light on the evolution of these small mammals on Earth.

## 1. Introduction

The genus of shrews *Suncus* (family Soricidae) comprises 18 species that are broadly distributed in Africa, Europe, and Asia, according to the most recent systematics re-evaluation (*Palawanosorex ater* is elevated from the genus *Suncus*) [1,2]. This genus *Suncus* (four upper unicuspids) can be distinguished from the nearest genus *Crocidura* (three upper unicuspids) using a dental formula [3]. A total of five *Suncus* species, Day’s Shrew *Suncus dayi*, Etruscan Shrew *Suncus etruscus*, Asian Musk Shrew *Suncus murinus*, Indian Highland Shrew *S. niger*, and Anderson’s Shrew *Suncus stoliczkanus* are inhabiting India; among them, *S. dayi* and *S. niger* are endemic to the country. The Indian Highland Shrew, *S. niger* with type locality = Madras in southern India [4] was treated as a subspecies of *Sorex montanus* (=*Suncus montanus*) [5], based on the external morphology [1,6]. The most current systematics evaluation distinguished these two species, *S. niger* for the Indian populations and *S. montanus* for the Sri Lankan populations [7,8]. However, these studies were restricted to previously generated genetic data of the *Suncus* species known as *S. montanus* requiring reevaluation of topotypes from its original distribution.

In addition to traditional taxonomy, molecular methods have been extensively used to assess the systematics, evolutionary relationships, and zoogeographic genetic diversity of *Suncus* species in Africa, the Middle East, and Southeast Asia [2,7,8,9,10,11,12,13,14,15,16,17]. Particularly the mitochondrial genes (Cytb and 16S rRNA) were found to be useful in re-evaluating the phylogeny of *Suncus* species, mainly focusing on groups from Western Asia, Southeast Asia, and Africa [18,19,20,21,22,23,24,25,26]. Nonetheless, the population genetic structure and phylogeography of *S. murinus* were disclosed based on mitochondrial gene variations and protein electrophoresis from various localities in South Asia and Southeast Asia. [27,28,29]. In addition to partial loci and multiple loci, the entire mitogenome of *Suncus* was also examined to determine the genomic organization, as well as the evolutionary trend of placental mammals [30,31]. 

Apart from the systematics research, the IUCN SSC Small Mammal Specialist Group (SMSG) assessed and categorized the status of *S. montanus* in both Sri Lankan and Indian (*S. niger*) as vulnerable due to significant risks such as habitat loss, farming growth, pesticide use, and forest fires. Although the IUCN SSC SMSG has not individually assessed the severely endangered *S. niger* since 2008, it is extremely important to protect this endemic species in the Western Ghats biodiversity hotspot. Six priority regions (Cameroon, Albertine Rift, Tanzania, Ethiopia, South Western Ghats in India, and Sri Lanka) were identified as globally threatened eulipotyphlans, which comprise 39.5% of all extant eulipotyphlan species (18.2% critically endangered, 41.0% endangered, and 46.2% vulnerable) and 17.6% of all species [32]. This area (Western Ghats biodiversity hotspot) has also been designated as a UNESCO World Heritage Natural Site due to its extraordinary geo-physical features and tropical environment; therefore, special attention is needed to protect the living biodiversity of this biogeographic region. Hence, the current research aimed to investigate *S. niger* from its original distribution in Southern India, combining morphology and genetic data to re-evaluate its systematics classification and distribution, evolutionary relationship, and potential diversification. Hence, the current research aimed to investigate *S. niger* from Southern India, combining morphology and genetic data to re-evaluate its systematics classification and distribution, evolutionary relationship, and diversification. We are confident that such research will aid in species recognition and range distribution, genetic diversity estimation, and the development of exact conservation strategies for the studied species and other non-volant animals in the near future.

## 2. Materials and Methods

### 2.1. Sampling and Morphological Analyses

**Figure 1:**
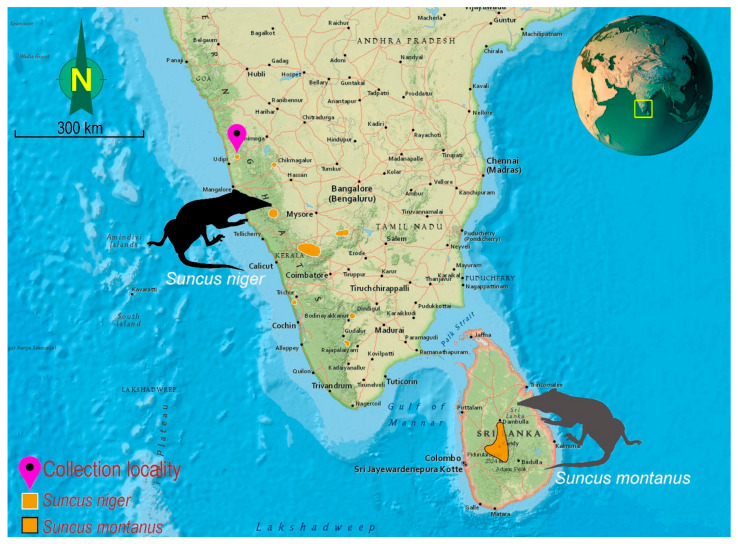
Map showing the distribution pattern of *S. niger* and *S. montanus* in India and Sri Lanka. Pink pin showing the collection locality and range extension of *S. niger* in the Western Ghats biodiversity hotspots in India.

Three adult males of *Suncus niger* were captured near Hulikal camp, Mookambika Wildlife Sanctuary, Udupi, Karnataka, India (13.725 N 75.010 E) through a pitfall method (Figure 1). Due to the nocturnal and secretive behavior of *Suncus* species and their vulnerability, three wild living specimens were used for this study. The external measurements were taken in the field, including length of head and body (HBL), length of tail (TL), height of ear (EH), and length of hindfoot excluding claw (HFL), as per standard method [33]. The craniodental measurements (GL: greatest length of skull; BL: basal length; CL: condylobasal length; MTR: length of maxillary tooth row; PL: palatal length; LR: length of rostrum; BB: breadth of braincase; PW: breadth of palate between the buccal margins of second molars; HB: height of braincase; ML: mandible length; LDT: length of dentary teeth excluding incisors; and DD: depth of dentary) were taken after skull extraction [33]. The morphological analyses of *S. niger* were performed as per standard protocol and the external and craniodental measurements were compared with other closely related species from the available museum specimens at the National Zoological Collections of the Zoological Survey of India (*S. murinus*: 16523, 16531, 16536, 16533, 16613 and *S. stoliczkanus*: 16217, 16218, 21398, 21399) and previous studies [8,22,34,35]. Furthermore, due to the lack of relevant biological materials, only a few morphological measurements (HBL, TL, EH, and HFL) of *S. zeylanicus* could obtain from the Global Biodiversity Information Facility (GBIF) database (https://www.gbif.org/species/2435505, accessed on 6 June 2023). Both tissue samples and skulls were vouchered in the National Zoological Collections of the Western Ghat Regional Centre (WGRC), Zoological Survey of India (ZSI), Kozhikode, India, under the registration numbers ZSI/WGRC/V.3624, ZSI/WGRC/V.3636, and ZSI/WGRC/V.3637. The experimental protocols were approved by the host institutions and were exhibited in accordance with relevant guidelines in compliance with ARRIVE 2.0. Guidelines [36].

### 2.2. DNA Extraction and Amplification

The total genomic DNA was extracted from the muscle tissues of three specimens of *S. niger* by the standard phenol–chloroform isoamyl alcohol method [37]. The primer pairs, mcb 398 (5′-TACCATGAGGACAAATATCATTCTG-3′) and mcb 869 (5′-CCTCCTAGTTTGTTAGGGATTGATCG-3′) and L2510 (5′-CGCCTGTTTATCAAAAACAT-3′) and H3059 (5′-CCGGTCTGAACTCAGATCACGT-3′), were used to amplify a partial segment of Cytb and 16S rRNA genes [38,39]. The 30 mL PCR mix contains 10 pmol of each primer, 20 ng of template DNA, 1X PCR buffer, 1.0–1.5 mM of MgCl2, 0.25 mM of each dNTP, and 1 U of High-fidelity Platinum Taq DNA Polymerase (Invitrogen, Life Science Technologies). The PCR reaction was executed in Veriti Thermal Cycler (Applied Bio systems, Foster City, CA, USA) with the standard thermal profile. The PCR products were cleaned using a QIAquick Gel Extraction Kit (QIAGEN Inc., Germantown, MD, USA) with the usual protocol. The cycle sequencing was performed by using BigDye Terminator ver. 3.1 Cycle Sequencing Kit (Applied Biosystems, Foster City, CA, USA). The bi-directional Sanger sequencing was accomplished by the Genetic Analyzer (Applied Biosystems) housed at Rajiv Gandhi Centre for Biotechnology (RGCB), Thiruvananthapuram, Kerala, India.

### 2.3. Sequence Quality Check and Dataset Preparation

Both forward and reverse chromatograms were screened through the SeqScanner Version 1.0 (Applied Biosystems) to avoid the noisy part of each sequence. The consensus sequences were further checked through nucleotide BLAST search (https://blast.ncbi.nlm.nih.gov/Blast.cgi, accessed on 6 June 2023) and ORF finder (http://www.ncbi.nlm.nih.gov/gorf/gorf.html, accessed on 6 June 2023) to validate in comparison to the vertebrate amino acid sequence array. New sequences were deposited in the GenBank database (Table S1). Additionally, a total of 42 Cytb sequences of other *Suncus* species were gathered from GenBank for comparative analyses. Further, 21 16S rRNA sequences of *S. murinus* and *S. montanus* were also acquired from GenBank for dual confirmation (Table S1). Due to the incorrect annotation, we did not include the previously generated sequence of *S. montanus* (EF524776) in the present analyses [16]. The DNA sequences of *Crocidura attenuata* (MW815430) were used as outgroup taxa in the present analyses. The generated and database sequences were by ClustalX software to form two datasets [40]. The Kimura-2-parameter (K2P) genetic distances were calculated in MEGAX for both Cytb and 16S rRNA datasets [41]. 

### 2.4. Phylogenetic Analyses and Time Tree

To perform the phylogenetic analyses, two different datasets were constructed with Cytb and 16S rRNA genes, respectively. The best-fit models were estimated by using JModelTest v2 with the lowest BIC (Bayesian information criterion) scores [42]. The Bayesian inference topologies were built by Mr. Bayes 3.1.2 by choosing nst = 6 with one cold and three hot chains (Metropolis-coupled Markov chain Monte Carlo). The program was run for 10,000,000 generations with tree sampling at every 100 generations with 25% of samples rejected as burn-in [43]. The topologies were edited by iTOL v6.8 webserver (https://itol.embl.de/login.cgi, accessed on 6 June 2023) [44]. To estimate the divergence time, the TimeTree analysis was performed using the RelTime method [45,46]. The baseline maximum likelihood (ML) tree was built in MEGA X with TN93 + G + I substitution model (lowest BIC score) [47]. The TimeTree was computed with a single calibration constraint of the split between the basal African major *Suncus* clade and the Eurasian *Suncus* that occurred 10.8 MYA ago (range 7.6–14.0) as ensued in a previous study [9]. 

## 3. Results 

### 3.1. Morphological Evidence

**Figure 2:**
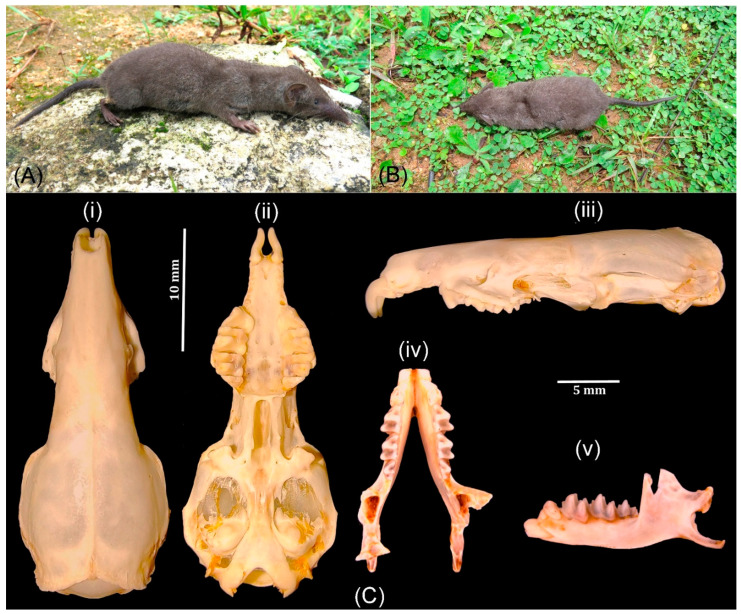
(**A**) Dorsolateral and (**B**) dorsal view of adult *S. niger* (photos taken by the second author, M.K.). (**C**) Cranium and mandible of *S. niger*: (**i**) dorsal, (**ii**) ventral, (**iii**) lateral views of the cranium, and (**iv**) occlusal and (**v**) lateral views of the mandible. (Note: the first four unicuspid teeth of the upper jaw and incisor of the lower jaw, and the condylar process of the left side mandible were unintendedly damaged during skull extraction.)

**Figure 3:**
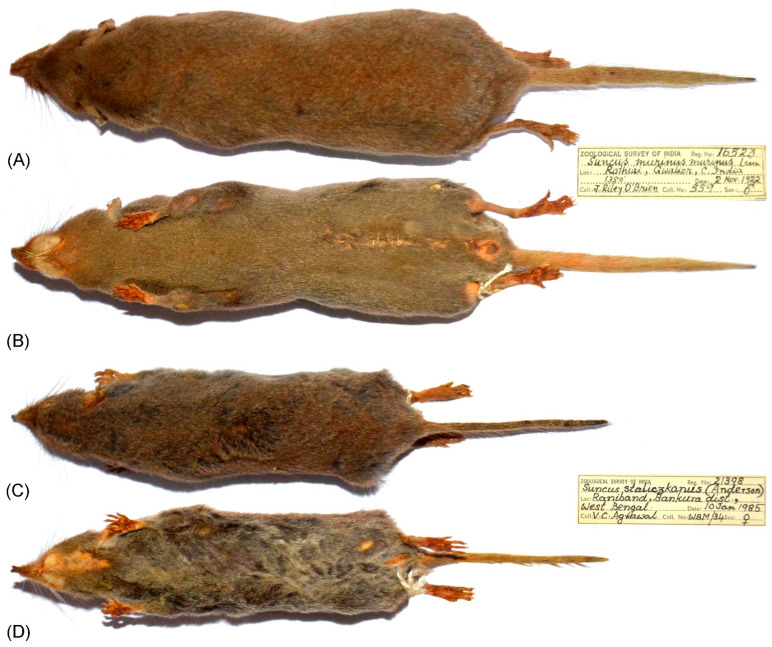
Dorsal and ventral views of *Suncus murinus* (**A**,**B**) and *S. stoliczkanus* (**C**,**D**) housed in the National Zoological Collections (Mammal and Osteology Section) of Zoological Survey of India, Kolkata.

The external and cranial measurements of *S. niger* (HBL: 80–110 mm) allow us to differentiate it from its congeners distributed in India and Sri Lanka. *Suncus niger* is smaller than *S. montanus* (HBL: 87.3–121.5 mm), *S. zeylanicus* (HBL: 108–114 mm), and *S. murinus* (HBL: 100–160 mm), whereas it is larger than *S. dayi* (HBL: 70–78 mm), *S. etruscus* (HBL: 42.1–42.4 mm), *S. fellowesgordoni* (HBL: 44–49.4 mm) and *S. stoliczkanus* (HBL: 68–85 mm) (Table 1). *S. niger* is comparatively smaller in the length of head and body, tail, hindfoot, and craniodental characteristics than *S. montanus*, though there is overlap to some degree between the two species (Table 1). Further, all dimensions of external and craniodental characters show that *S. niger* is a larger size than *S. stoliczkanus* and considers two different species morphologically (Table 1, Figure 2 and Figure 3). In addition to the external morphometrics, *S. niger* has a blackish body throughout compared with other congeners.

### 3.2. Molecular Identification and Genetic Divergence

In the partial Cytb gene (427 bp), the overall mean genetic distance of the present dataset of *Suncus* species was 14.3%. Our specimens of *S. niger* showed 98.29–99.29% similarity with *S. niger* (*S. montanus*) (DQ630388, from Kotagiri, Tamil Nadu, India) through nucleotide BLAST search. The Cytb sequences comprise an average of 58.89% AT composition in *Suncus* species. A total of 38% variable sites were detected in the partial Cytb sequences among *Suncus* species, while 2% variable sites were found in *S. niger*. The targeted species, *S. niger* showed substantial genetic distances from other *Suncus* species ranging from 8.49% (*S. stoliczkanus*) to 26.29% (*S. hututsi*). *Suncus niger* also maintained an 8.66% genetic distance from *S. montanus*, which was considered the most closely related species. The intra-species genetic distance ranged from 0% to 1.65%, except for *S. murinus*. An exceptionally high intra-species genetic distance (3.68%) was observed in *S. murinus*. The inter-species genetic distance ranged from 3.15% to 26.29% (Table 2). 

The genetic distinctiveness of *S. niger* was further tested by the mt 16S rRNA gene. The generated 16S rRNA sequence showed 99.08–99.82% similarity with *S. montanus* (DQ630304 and EF524884) from Tamil Nadu, India through nucleotide BLAST search. The 16S rRNA sequences (580 bp) comprise an average of 59.3% AT composition in all *Suncus* species with 58.9–59.2% in *S. niger*. A total of 6% variable sites were detected in the partial 16S rRNA gene among all *Suncus* species, while 1.2% variable sites were found in *S. niger*. The overall mean genetic distance (16S rRNA) of all three species was 1.9%. In the 16S rRNA gene, *S. niger* showed 3.37% and 3.39% genetic distance with *S. montanus* and *S. murinus*, respectively (Table 2). The intra-species genetic distance ranged from 0.52% (*S. niger*) to 1.26% (*S. montanus*). Overall, both Cytb and 16S rRNA sequences clearly discriminated *S. niger* from other *Suncus* species with high genetic divergence. 

### 3.3. Matrilineal Phylogeny and Divergence Time

**Figure 4:**
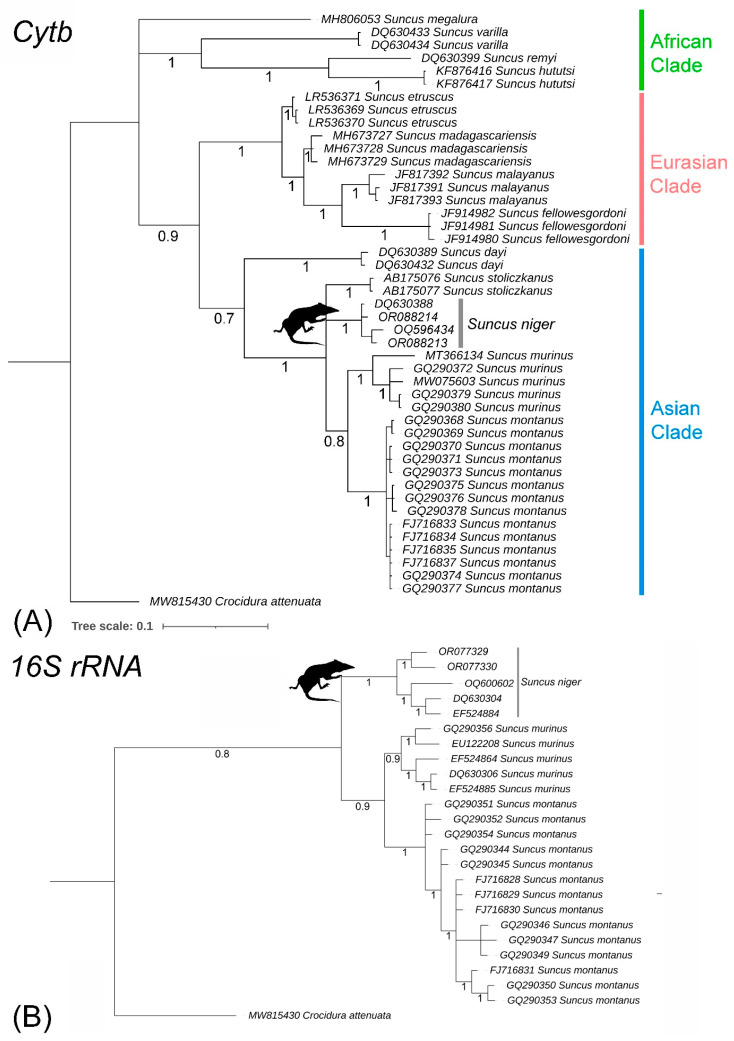
(**A**) The Bayesian phylogeny based on (**A**) Cytb and (**B**) 16S rRNA genes clearly delineated *S. niger* from other congeners. The posterior probability supports are represented by values with each branch.

The Cytb and 16S rRNA genes unambiguously differentiated all *Suncus* species in the Bayesian phylogeny (Figure 4). Our sequences of *S. niger* showed distinct monophyletic clustering in both mitochondrial genes. *Suncus niger* maintained a close connection with *S. stoliczkanus* in the mtCytb tree, as shown in the previous study [7,25]. According to both Cytb and 16S rRNA species trees, *S. niger* early split from the basal node of *S. montanus* and *S. murinus* (Figure 4A,B). The mtCytb-based BA topology showed three major clades of *Suncus*: African clade (*S. megalura*, *Suncus varilla*, *Suncus remyi*, and *S. hututsi*), Eurasian clade (*S. etruscus*, *S. madagascariensis*, *S. malayanus*, and *S. fellowesgordoni*), and Asian clade (*S. dayi*, *S. stoliczkanus*, *S. niger*, *S. murinus*, and *S. montanus*) (Figure 4A). Except for *S. etruscus* and *S. madagascariensis* species complex, other *Suncus* species showed genetic distances between 3.68% to 7.77% in the mtCytb gene. 

**Figure 5:**
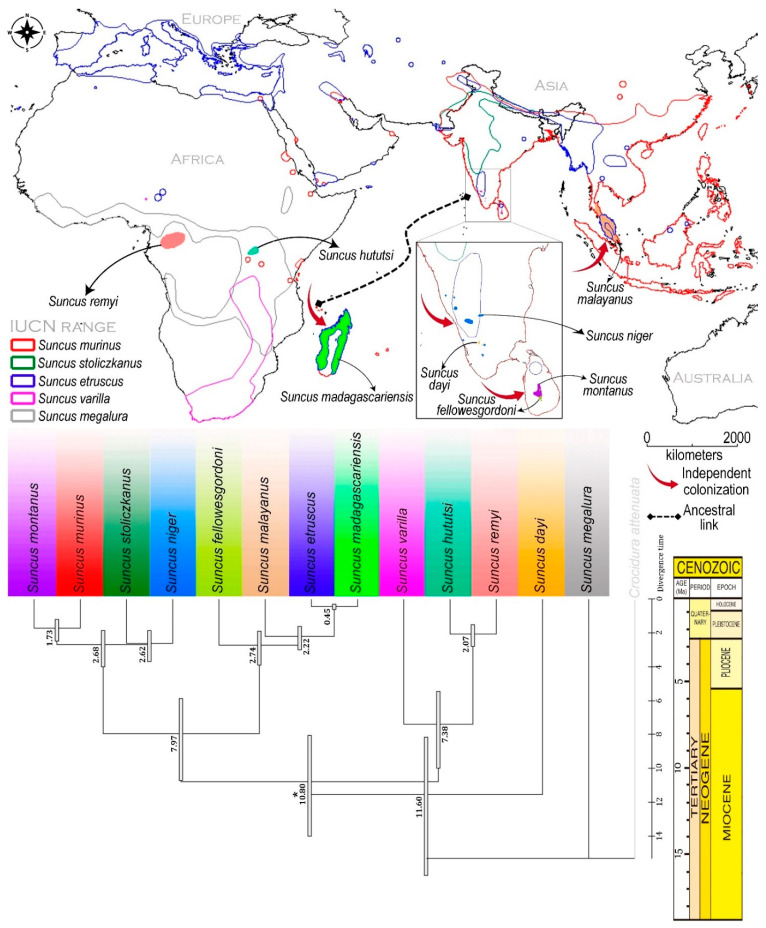
Map showing the colonization of *Suncus* species during late Miocene to the Holocene (**above**). The maximum likelihood topology with relative times showed the approximate divergence time of the *Suncus* species (**below**). The TimeTree was computed with the calibration constraint (marked by an asterisk) determined from the previous study [9].

The current ML-based RelTime analysis in the Cytb gene revealed a similar topology with Bayesian inference, which is consistent with previous studies [9,10]. The TimeTree showed that *S. niger* diverged during the late Pliocene (≈2.62 MYA) (Figure 5). According to the current mitochondrial gene-based relaxed molecular clock, *S. megalura* is the most distantly related species to all *Suncus* members. On the other hand, the Asian species, *S. dayi* (endemic to India), showed significant affinity towards African species and separated from other *Suncus* during the Miocene (≈11.60 MYA) (Figure 5). 

## 4. Discussion

The actual number of animal extinctions over the last 100 years informs us of the continuing worldwide sixth mass extinction caused by anthropogenic activity, climate change, and ecological decline [48,49]. As a result, life protection is a priority in order to support ecosystem and human well-being via a new unified idea and the execution of worthwhile conservation strategies [50]. Currently, evaluation teams are highly skewed toward launching numerous conservation initiatives for higher vertebrate and/or charismatic species. In this situation, we are pushing many putative species towards extinction before we even realize it [51,52]. In the zoological study, taxonomic confirmation is critical for any name-bearing taxa. Hence, incorporating molecular methods, in addition to conventional morphometric measurements, is now a fundamental prerequisite of systematics research. Such combined knowledge also aids in assessing the status of any species and developing conservation action plans to keep it in its ecosystem.

Small mammals, such as non-volant species, are less concentrated groups in terms of evaluation and protection than larger mammals. Among all Soricomorphs, *Suncus* is one of the barely studied groups compared to other groups such as *Crocidura*, requiring in-depth taxonomic assessment across their vast zoogeography [25]. The morphological characteristics of *Suncus* species often overlap, leading to misunderstandings during taxonomic evaluation. To address these issues, machine learning methods were recently employed to differentiate the *S. murinus* species complex in Peninsular Malaysia [53]. Prior to this study, although the identity and systematics of the Indian endemic eutherian (*S. niger*) were well accepted [7,8,54,55], it was necessary to reassess this taxon from its type locality. Both morphometric and molecular data verified the species status of *S. niger* in the current research, which is consistent with the prior hypothesis [7,8,25]. The external appearance of *S. niger* is mostly similar to the Asian Musk Shrew (*S. murinus*), but distinguished by the overall blackish body including its hands, feet, tail, ears, and muzzle, and relatively smaller. *S. niger* has a dark slender tail with black hairs and is extremely docile, and non-squeaky unlike *S. murinus*; with regards to habitat differentiation, *S. niger* is a forest-dwelling species and *S. murinus* lives near human habitation [34]. Except for the body color and habitat, *S. niger* is previously evidenced as a distinct species based on the differences in size in comparison with its relative species [1,6]. To corroborate the taxonomic identity of *S. niger*, both external and craniodental parameters were measured and compared with the closely related species (*S. dayi*, *S. etruscus*, *S. fellowesgordoni*, *S. montanus*, *S. murinus*, and *S. stoliczkanus*) distributed in India and Sri Lanka [8]. In small mammals, morphology alone cannot discriminate; therefore, discrete morphological characters are needed to investigate them at the species level. Furthermore, the molecular data clearly separated *S. niger* from *S. montanus* (restricted to Sri Lanka) and other species distributed in Asia, Africa, and Europe, which is consistent with pre-suggested genetic distance (≥8%) as significant for elucidating species level discrimination [56,57].

The separation of India–Madagascar–Seychelles from the African plate occurred during the Middle Jurassic through the latest Eocene (166–35 MYA) [58], long before soricids evolved. However, the evolutionary relationship of Asian *Suncus* (*S. dayi*) and African *Suncus* (*S. varilla*, *S. hututsi*, *S. remyi*, and *S. megalura*) hints at an ancestral link between these continents prior to prehistoric Gondwana vicariance, and that both landmasses exchanged ancestral genetic lineages that later evolved into distinct species through independent transcontinental colonization as depicted in previous studies [59,60]. However, we could not rule out the alternative explanation that the ancestor of soricids diverged before the separation of Laurasia and Gondwana and that both African and Asian lineages were later confined, respectively. Both Indian and Sri Lankan large-bodied mobile mammals are genetically similar, and their diversification has been significantly influenced by plate tectonic events following Pangea’s split and reconnection with Asia after the Paleocene [61]. However, the evolutionary pattern of Indian (endemic to Western Ghats) and Sri Lankan animals is debatable, with modern Indian mammals evolving through the ‘out-of-India’ or ‘out-of-Asia’ hypothesis [61,62,63]. The strong link between *S. niger* and *S. stoliczkanus* in the present TimeTree indicates that their diversification occurred independently on the Asian continent at the same time. This baseline data will spur further research using mitochondrial and nuclear genetic markers to determine the actual diversification of *Suncus* species. Furthermore, there is evidence of multiple populations of the Asiatic house shrew (*S. murinus*) and the Etruscan shrew (*S. etruscans*) spanning different continents (Africa, Europe, and Asia) and insular islands [23,24,27,28,29,64]. Hence, large-scale investigations using an integrated approach are needed for these two species, especially for Southern Europe and Southeast Asian countries, to better understand the hidden diversity and spectacular island radiations as observed in other Soricids species [65].

In terms of habits and habitats, *S. niger* is nocturnal, crepuscular, semi-fossorial, and lives far from human settlement [66,67]. This species is restricted to the wet, humid, and montane forests of southern India’s Western Ghats (Nilgiris, Palani hills, Coorg), at elevations ranging from 900 to 2400 m asl [66,68]. In a tropical montane environment in the Western Ghats, this endemic shrew prefers habitats with higher tree density and ground cover, and lower canopy height [69]. Although distinct patterns of various environments are evident, microhabitat selection and segregation are limited in these small animals, which may be influenced by intraspecific competition in these groups [69]. Based on the external morphology, a recent study recorded *S. niger* from the Bababudan Hills (13.437945 N 75.758163 E, 1707 m asl) in the state of Karnataka, India [54]. However, the current record at Mookambika Wildlife Sanctuary (85 km northwest of the latest record) in the state of Karnataka provides a new range extension and the lowest elevation (600 m asl) habitat of *S. niger*. This sanctuary encompasses tropical evergreen forests semi-evergreen forests, wet mixed deciduous forests, and dry grasslands (Government of Karnataka-Forest Department: Management Plan, Mookambika Wildlife Sanctuary, https://aranya.gov.in/downloads/Kollur_MgmtPlan.pdf, accessed on 6 June 2023). Thus, the current finding demonstrates the occurrence of *S. niger* in a wet mixed deciduous forest at the lowest elevation as well as inside and outside of the protected areas, which has to be investigated further. Aside from climate change, a slew of other threats, such as forest fragmentation, agricultural expansion, human encroachment, and road construction, put a strain on the current population of small mammals in this region [70,71]. The data provided here will aid in the evaluation of this restricted-range mammal species in the near future, as well as decipher information to support conservation action plans to safeguard them in the wild. The current research further promotes population structure studies of this vulnerable mammal in the insular habitat of the Western Ghats Biodiversity hotspot to protect its endemism and evolutionary potential.

## 5. Conclusions

The current research re-evaluates the species status of the Indian Highland Shrew, *S. niger*, from its type locality in the Western Ghats biodiversity hotspot in India. *Suncus niger* was readily delineated from other congeners based on morphometric and molecular data (Cytb and 16S rRNA genes). The present study also provides new range expansion and height records for this small mammal. The genetic distance and phylogeny are consistent with the genetic species concept for mammals. The present study reasserted that *S. niger* is genetically related to *S. stoliczkanus* rather than *S. montanus*. The estimated divergence times and evolutionary trends reveal that the ancestral genetic lineages of *Suncus* may have dispersed from Africa to other continents, and that these genetic components later differentiated into separate species by independent transcontinental colonization. However, we believe that large-scale efforts in taxonomic, ecological, and genomics research are required to fully understand the diversity, population structure, and evolutionary linkages of *Suncus* species throughout the world. Such a combined strategy will also aid in the development of effective conservation action plans for these small mammals in their environment.

## Figures and Tables

**Table 1:** External morphology and craniodental measurements (in mm) of *S. niger* and other congeners distributed in India and Sri Lanka. The comparative measurements of closely related species were obtained from the ZSI, previous studies [8,22,34,35], and the GBIF database. Abbreviations for measures are shown in Materials and Methods. *S. niger* = *Sn*; *S. montanus* = *Smo*; *S. murinus* = *Smu*; *S. etruscus* = *Se*; *S. fellowesgordoni* = *Sf*; *S. dayi* = *Sd*; *S. stoliczkanus* = *Ss*; *S. zeylanicus* = *Sz*; *n* = number of specimens; measurements are minimum to maximum.

Measurements	*Sn* [This Study]	*Sn* [34]	*Smo* [8]	*Smu* [8]	*Smu* [ZSI]	*Se* [22]	*Sf* [22]	*Sd* [35]	*Ss* [35]	*Ss* [ZSI]	*Sz* (GBIF)
	India	India	Sri Lanka	Sri Lanka	India	Sri Lanka	Sri Lanka	India	Sri Lanka	India	Sri Lanka
	(*n* = 3)	(*n* ≥ 1)	(*n* = 18)	(*n* = 8)	(*n* = 5)	(*n* = 3)	(*n* = 5)	(*n* = 7)	(*n* = 11)	(*n* = 4)	(*n* = 2)
HBL	80–110	80–105	87.3–121.5	100–160	107–120	42.1–42.4	44.0–49.4	70–78	68–85	59–75	108–114
TL	45–63.51	45–65	56.1–75.5	72–85	62–69	27.4–31.1	33.5–38.0	83–88	44–55	37–54	92–97
EH	11.2–12.69	-	8–12.6	11.4–13.6	0.9–13	5.9–6.2	5.6–7.0	-	-	0.9–10	10–13
HFL	14–18	14–17	15.6–19.1	20.1–23.4	16–18.5	7.4–7.6	9.6–11.0	15.5–16.5	10.5–15	11–14	20
GL	26.3–27.3	-	23.1–27.8	27–34.3	24–27.3	11.9	12.9–13.7	-	-	14.4–15.2	-
BL	22.9–24.2	-	20.5–25.3	24.3–31.7	21.2–25.6	11.0–11.1	11.7–12.3	-	-	13.5–14.2	-
CL	26–27.5	-	22.9–27.8	27–34.7	24.1–28.7	12.0–12.1	12.7–13.6	18.9–20.2	18.6–22.2	18.6–22.2	-
MTR	6.9–7.9	-	6.8–8.1	7.5–9.4	6.9–7.8	2.8–3.1	3.6–3.9	8.5–8.8	8.1–10.3	6.1–6.6	-
PL	11.6–12.2	-	10.2–12.5	11.8–15.5	10.8–12.5	3.8–4.5	4.8–5.4,	-	-	8.2–9.1	-
LR	9.5–10.1	-	8.4–10.3	10.2–12.4	9.8–11.9	3.8–4.0	4.2–4.7	-	-	6.7–6.5	-
BB	11.3–12.1	-	9.7–12.3	11.3–15.3	10.7–13.3	5.3	5.5–5.7	8.3–9.8	8.9–9.6	8.9–9.6	-
PW	6.9–7.4	-	6.4–7.7	8.2–9.5	8.1–8.9	3.0–3.1	3.3–3.9	5.6–6.0	5.2–6.9	5.2–6.9	-
HB	5.8–6.1	-	5.4–6.5	6.3–7.9	6.2–7.5	2.5–2.6	3.0–3.2	5.0–5.3	3.9–5.0	3.9–5.0	-
ML	13.6–14.7	-	13.2–15.3	14.7–19.1	13.7–15.2	6.0–6.3	6.9–7.3	-	-	10.5–11.2	-
LDT	7.0–8.0	-	7.0–8.4	8.1–9.8	7.8–8.3	3.0–3.2	3.8–3.9	-	-	6.1–6.7	-
DD	6.4–7.7	-	5.9–8.5	8–11	7.9–9.1	2.7–3.0	3.2–3.5	-	-	5.6–5.8	-

**Table 2:** Inter-species and intra-species Kimura-2-parameter (K2P) genetic divergence of *Sucus* species based on mtCytb. The intra-species genetic distance of *S. megalura* and *S. remyi* is not calculated (n/c) due to single sequence.

Species	Inter-Species												Intra-Species
*S. niger*													1.14
*S. montanus*	8.66												1.65
*S. murinus*	9.85	7.87											3.68
*S. stoliczkanus*	8.49	9.50	9.67										0.24
*S. malayanus*	15.78	14.59	15.32	17.25									1.47
*S. madagascariensis*	16.17	16.42	16.14	18.08	7.31								0.97
*S. etruscus*	16.30	15.48	16.50	17.66	8.66	3.15							0.16
*S. fellowesgordoni*	16.45	18.12	17.34	18.27	9.85	10.83	12.24						0.32
*S. dayi*	18.19	19.20	19.42	17.50	21.52	18.02	15.86	22.30					0.73
*S. varilla*	21.18	21.22	22.23	22.66	22.15	20.17	17.88	20.07	22.06				0.00
*S. megalura*	21.85	19.83	19.61	21.07	27.03	23.88	23.18	26.19	19.23	22.60			n/c
*S. remyi*	24.63	23.66	24.59	22.03	21.71	22.33	19.69	21.37	22.17	20.69	25.72		n/c
*S. hututsi*	26.29	24.40	25.45	24.93	22.88	24.36	24.49	24.56	21.58	23.44	21.86	14.11	0.00

## Data Availability

The nucleotide sequence data that support the findings of this study are openly available in GenBank of NCBI at [https://www.ncbi.nlm.nih.gov] under accession Nos. OQ596434, OR088213, and OR088214 (Cytb) and OQ600602, OR077329, and OR077330 (16s rRNA).

## Supplementary Materials

The following supporting information can be downloaded at: https://www.mdpi.com/article/10.3390/genes14071493/s1, Table S1. Details of the generated and GenBank sequences of S. species used in the present datasets [72,73].
