# Supplementary material for: Morphology and Mitochondrial Lineage Investigations Corroborate the Systematic Status and Pliocene Colonization of Suncus niger (Mammalia: Eulipotyphla) in the Western Ghats Biodiversity Hotspot of India
Source: Genes (Basel). 2023 Jul 22;14(7):1493. doi: 10.3390/genes14071493 (PMC10379166; doi:10.3390/genes14071493)
Supplement: Supplementary file 1 [file genes-14-01493-s001.zip › genes-2478082-supplementary.pdf]

Table S1. Details of the generated and GenBank sequences of *S.* species used in the present datasets.

Species	Gene	Accession No.	Voucher No.	Locality	Reference
S. niger	Cytb	OQ596434	ZSI/WGRC/V.3624	India	This Study
S. niger	Cytb	OR088213	ZSI/WGRC/V.3636	India	This Study
S. niger	Cytb	OR088214	ZSI/WGRC/V.3637	India	This Study
S. montanus	Cytb	DQ630388	IZEA V573	India	[9]
S. megalura	Cytb	MH806053	VN1416/MNHN-ZM-2013-571	Guinea	Nicolas et al. 2019
S. varilla	Cytb	DQ630433	IZEA 3	South Africa	[9]
S. varilla	Cytb	DQ630434	IZEA 4	South Africa	[9]
S. remyi	Cytb	DQ630399	SBP GA3650	Gabon	[9]
S. hututsi	Cytb	KF876416	FMNH207302	Rwanda	Demos et al. 2014
S. hututsi	Cytb	KF876417	FMNH 207303	Rwanda	Demos et al. 2014
S. etruscus	Cytb	LR536371	TAUM14251	Israel	[25]
S. etruscus	Cytb	LR536369	TAUM14397	Israel	[25]
S. etruscus	Cytb	LR536370	TAUM14402	Israel	[25]
S. madagascariensis	Cytb	MH673727	4775	Iran	[17]
S. madagascariensis	Cytb	MH673728	4776	Iran	[17]
S. madagascariensis	Cytb	MH673729	saAM	Iran	[17]
S. malayanus	Cytb	JF817392	ZO-SMAL(CH)-02	Malaysia	[20]
S. malayanus	Cytb	JF817391	ZO-SMAL(PP)-01	Malaysia	[20]
S. malayanus	Cytb	JF817393	ZO-SMAL(BR)-03	Malaysia	[20]
S. fellowesgordoni	Cytb	JF914982	WHT 6819	Sri Lanka	[15]
S. fellowesgordoni	Cytb	JF914981	WHT 6818	Sri Lanka	[15]
S. fellowesgordoni	Cytb	JF914980	WHT 6817	Sri Lanka	[15]
S. dayi	Cytb	DQ630389	IZEA V567	India	[9]
S. dayi	Cytb	DQ630432	IZEA V576	India	[9]
S. stoliczkanus	Cytb	AB175076	HA7268	Nepal	[19]
S. stoliczkanus	Cytb	AB175077	HA7270	Nepal	[19]
S. murinus	Cytb	MT366134	NPAR0587	Nepal	GenBank
S. murinus	Cytb	GQ290372	WHT6907	Sri Lanka	[8]
S. murinus	Cytb	MW075603	S3717	China	GenBank
S. murinus	Cytb	GQ290379	WHT6924	Sri Lanka	[8]
S. murinus	Cytb	GQ290380	WHT6928	Sri Lanka	[8]
S. montanus	Cytb	GQ290368	WHT 6796	Sri Lanka	[8]
S. montanus	Cytb	GQ290369	WHT6815	Sri Lanka	[8]
S. montanus	Cytb	GQ290370	WHT6816	Sri Lanka	[8]
S. montanus	Cytb	GQ290371	WHT6822	Sri Lanka	[8]
S. montanus	Cytb	GQ290373	WHT6855	Sri Lanka	[8]
S. montanus	Cytb	GQ290375	WHT6848	Sri Lanka	[8]
S. montanus	Cytb	GQ290376	WHT6850	Sri Lanka	[8]
S. montanus	Cytb	GQ290378	WHT6841	Sri Lanka	[8]
S. montanus	Cytb	FJ716833	WHT6860	Sri Lanka	[7]
S. montanus	Cytb	FJ716834	WHT 6840	Sri Lanka	[7]
S. montanus	Cytb	FJ716835	WHT 6852	Sri Lanka	[7]
S. montanus	Cytb	FJ716837	WHT 6814	Sri Lanka	[7]
S. montanus	Cytb	GQ290374	WHT 6847	Sri Lanka	[8]
S. montanus	Cytb	GQ290377	WHT6851	Sri Lanka	[8]
S. niger	16s rRNA	OQ600602	ZSI/WGRC/V.3624	India	This Study
S. niger	16s rRNA	OR077329	ZSI/WGRC/V.3636	India	This Study
S. niger	16s rRNA	OR077330	ZSI/WGRC/V.3637	India	This Study
S. montanus	16s rRNA	DQ630304	IZEA V573	India	[9]
S. montanus	16s rRNA	EF524884	IZEAV559		[10]
S. murinus	16s rRNA	GQ290356	WHT6928	Sri Lanka	[8]

S. murinus	16s rRNA	EU122208	WHT 6906	Sri Lanka	[8]
S. murinus	16s rRNA	EF524864	LRH3544	Philippines	[10]
S. murinus	16s rRNA	DQ630306	ZEA V546	India	[9]
S. murinus	16s rRNA	EF524885	IZEAV554	India	[10]
S. montanus	16s rRNA	GQ290351	WHT6848	Sri Lanka	[8]
S. montanus	16s rRNA	GQ290352	WHT6850	Sri Lanka	[8]
S. montanus	16s rRNA	GQ290354	WHT6941	Sri Lanka	[8]
S. montanus	16s rRNA	GQ290344	WHT 6796	Sri Lanka	[8]
S. montanus	16s rRNA	GQ290345	WHT6815	Sri Lanka	[8]
S. montanus	16s rRNA	FJ716828	WHT6814	Sri Lanka	[7]
S. montanus	16s rRNA	FJ716829	WHT6860	Sri Lanka	[7]
S. montanus	16s rRNA	FJ716830	WHT6840	Sri Lanka	[7]
S. montanus	16s rRNA	GQ290346	WHT6816	Sri Lanka	[8]
S. montanus	16s rRNA	GQ290347	WHT6822	Sri Lanka	[8]
S. montanus	16s rRNA	GQ290349	WHT6855	Sri Lanka	[8]
S. montanus	16s rRNA	FJ716831	WHT6852	Sri Lanka	[7]
S. montanus	16s rRNA	GQ290350	WHT 6847	Sri Lanka	[8]
S. montanus	16s rRNA	GQ290353	WHT6851	Sri Lanka	[8]

Additional References:

72 Nicolas, V.; Jacquet, F.; Hutterer, R.; Konečný, A.; Kouassi, S.K.; Durnez, L.; Lalis, A.; Colyn, M.; Denys, C. Multilocus Phylogeny of the *Crocidura Poensis* Species Complex (Mammalia, Eulipotyphla): Influences of the Palaeoclimate on Its Diversification and Evolution. *J. Biogeogr.* **2019**, *46*, 871-883.

73 Demos, T.C.; Kerbis Peterhans, J.C.; Agwanda, B.; Hickerson, M.J. Uncovering cryptic diversity and refugial persistence among small mammal lineages across the Eastern Afromontane biodiversity hotspot. *Mol. Phylogenet. Evol.* **2014**, 71, 41-54.
